# Tumor-Associated Neutrophils in Cancer: Going Pro

**DOI:** 10.3390/cancers11040564

**Published:** 2019-04-19

**Authors:** Lingyun Wu, Sugandha Saxena, Mohammad Awaji, Rakesh K. Singh

**Affiliations:** Department of Pathology and Microbiology, University of Nebraska Medical Center, 985900 Nebraska Medical Center, Omaha, NE 68198-5900, USA; lingyun.wu@unmc.edu (L.W.); sugandha.saxena@unmc.edu (S.S.); mohammad.awaji@unmc.edu (M.A.)

**Keywords:** neutrophils, cancer metastasis, neutrophil extracellular traps, neutrophil polarization

## Abstract

The progression of cancer is not only about the tumor cell itself, but also about other involved players including cancer cell recruited immune cells, their released pro-inflammatory factors, and the extracellular matrix. These players constitute the tumor microenvironment and play vital roles in the cancer progression. Neutrophils—the most abundant white blood cells in the circulation system—constitute a significant part of the tumor microenvironment. Neutrophils play major roles linking inflammation and cancer and are actively involved in progression and metastasis. Additionally, recent data suggest that neutrophils could be considered one of the emerging targets for multiple cancer types. This review summarizes the most recent updates regarding neutrophil recruitments and functions in the tumor microenvironment as well as potential development of neutrophils-targeted putative therapeutic strategies.

## 1. Introduction

Cancer-related fatalities rank as the second leading cause of death in all ages and both genders in the United States [1]. Generally, the treatments for early-stage cancers are effective, contrary to late-stage when the malignancies spread to remote organs. Currently, the primary treatments for cancer include chemotherapy, radiation, and surgery [2]. The working mechanisms of chemotherapy drugs usually involve inhibiting DNA synthesis as well as cell replication, cell mitotic, or inhibiting topoisomerases [3]. However, cancer cells can easily develop therapy resistance through mechanisms including a decrease in drug intake, increase in drug efflux, or epithelia to the mesenchymal transition (EMT), and changes in the tumor microenvironment [4,5]. Through these mechanisms, tumor cells relapse and metastasize to distant organs even after treatment. Consequently, there is an urgent need for an effective therapeutic plan for cancer patients.

Previous studies demonstrate that the tumor microenvironment plays a crucial role in cancer metastasis [6]. The tumor microenvironment significantly affects the therapeutic response and the overall outcome of the patients. Additionally, neutrophils as a critical factor in the tumor microenvironment play an essential regulatory role in cancer progression [7].

Neutrophils, which originate from the myeloid precursor, compose the significant cellular parts of white blood cells and are the primary responsive cell type for an innate immune response [7,8]. Neutrophils, the hallmark of acute inflammation [8], are polymorpho-nuclear cells, which derives its name ‘neutrophil’ from the positive staining of both hematoxylin and eosin dyes. Based on traditional immunology, neutrophils are mainly responsible for host defense, immune modulation, and tissue injury [9]. Their functions in tumor progression were neglected due to these traditional ideas as well as their short survival time (around 3 to 24 h) [10]. However, recent results proved the phenotype heterogeneity and functional versatility of the neutrophils [7]. Researchers found that neutrophils play a pivotal role in chronic inflammatory diseases including cancer. They function in a more complex way when compared to traditional ideas about neutrophils [11]. Specifically, neutrophils can survive longer than researchers’ initial belief (5 or more days in circulation), especially in the tumor microenvironment [12]. There are some pro-inflammatory factors in the tumor microenvironment reported to extend neutrophil survival time such as the interferon gamma (INFγ) [13], and activate tumor-associated neutrophils in different conditions, which results in anti-tumor and pro-tumor functions of neutrophils [14]. Currently, there is increasing evidence indicating that neutrophils are playing vital functions in the tumor microenvironment [7]. However, the nature of these roles in different cancer types is still debatable [15]. Meanwhile, the population of neutrophils renders phenotypic heterogeneity and functional versatility. However, how to clarify the polarization states of the tumor-associated neutrophils is still under investigation. Additionally, the detailed mechanism regarding neutrophil-facilitated cancer progression remains unclear.

Under cancer and severe injury conditions, neutrophils are often associated with a granulocytic population of myeloid-derived suppressor cells (gMDSCs). gMDSCs share similar morphology and expression of cell surface markers with mature neutrophils, but the difference lies in the suppression capacity of T-lymphocytes [16]. This review summarizes the neutrophil recruitment, functions, and regulator of neutrophil functions in the tumor microenvironment. We will also discuss the clinical potential of using neutrophils as a prognostic marker, therapeutic target, and potential biomarker in cancer patients.

## 2. Neutrophil Recruitment in Cancer

The mobilization of neutrophils from bone marrow to tumor sites occurs in three phases including expansion and maturation of pre-mature neutrophils in the bone marrow, intravasation to circulation through attachment to endothelial cells, and the chemotactic movement of neutrophils to tumor sites [17]. The pre-mature neutrophils are derived from hematopoietic stem cells. The proliferation and maturation of neutrophils require the regulation of granulocyte colony-stimulating factor (G-CSF) and granulocyte macrophage colony stimulating factor (GM-CSF) [7]. The neutrophil maturation also includes the nuclear morphology change—the original round-shape nucleus finalizes to a segmented shaped nucleus and surface antigen expression changes including CD 65 and CD16 [18].

The release of neutrophils in bone marrow mainly depends on the interplay between CXCR4 and CXCR2 and its ligands [19]. These two receptors belong to the CXC chemokine receptor family as G-protein coupled receptors. CXCR4 and CXCR2 are expressed on the surface of the neutrophil and span seven times the neutrophil membrane [20]. The role of CXCR4 is for neutrophil homing in the bone marrow. Higher levels of CXCR4 and its ligands (for instance, CXCL12) will restrain the neutrophils mobility [19,21]. An initial step for neutrophil movement is the disruption of CXCR4 and its ligand expression by factors including G-CSF.

Conversely, the CXCR2 receptor is mainly responsible for the release of neutrophils into circulation, CXCR2, CXCR2 ligands, and G-CSF co-ordinates together to facilitate neutrophil mobilization [17]. Antagonistic interactions between CXCR2 and CXCR4 maintains the homeostasis of neutrophils [19,21]. The increased expression of CXCR2 indicates the mobilization of mature neutrophils to the circulation system. Meanwhile, the upregulation of CXCR4 on aged neutrophils will result in them backing to the bone marrow and being digested by macrophages [22].

The mobilization of neutrophils to the tumor sites also requires an interplay between CXCR2 and its ligands CXCL1-3 and CXCL5-8 [23,24]. In cancer, the CXCR2 axis is the primary player for neutrophil recruitment to the tumor sites [25]. Multiple cell types within the tumor produce the CXCR2 chemokines including tumor cells, immune cells, and cancer-associated fibroblasts [24,26,27]. Once the neutrophil mobilization is needed, these contributors will release the CXCR2 chemokines into the circulation system. The neutrophils will then move through a positive chemotactic gradient, towards the higher concentration of the CXCR2 ligands. The expression of CXCR2 on neutrophil cell surfaces and the production of CXCR2 ligands are both vital for this chemotactic movement [21,28]. The inhibition of CXCR2 expression in neutrophils will result in neutrophil retention in bone marrow [28]. Additionally, the inhibition of CXCR2 ligands also ends in significantly reduced neutrophil mobilization [29].

Based on previous reports, there are several positive regulators, which lead to an increase in neutrophil recruitment such as G-CSF and interleukin 17 (IL17). G-CSF is a cytokine produced by multiple cells types, including macrophage, endothelial cells [30], and cancer cells [31]. Apart from neutrophil mobilization, G-CSF is also known to play a role in neutrophil proliferation, maturation, and function [20,32,33,34]. G-CSF positively regulate neutrophil migration by downregulating the expression of CXCR4 and its ligand, CXCL12 [16]. Blocking the G-CSF receptor in mice ends up with impaired neutrophil mobilization [35]. However, experiments showed that G-CSF did not induce the chemotactic effects on murine neutrophils [16], which indicates G-CSF induced neutrophil migration mainly depend on the indirect activity of G-CSF. The recruitment of neutrophils seems to be more dependent on CXCR4, CXCR2, and their ligands [20].

Another critical factor for neutrophil recruitment is IL17 [36]. The IL17 family consists of six members, IL17A-F. Since IL17A is the most notable members in the family, it is also known as IL17. IL17 was found to upregulate the expression levels of various cytokines and chemokines, including G-CSF [37], IL6, CCL2 (MCP-1) [38], and CXCR2 ligands [39]. IL17 is positively linked with neutrophil numbers in the tumor microenvironment [36]. In breast cancer models, IL17 is found to increase the secretion of CXCL1 and CXCL5 by mammary carcinoma cells, which further facilitates cancer progression [40]. Consequently, higher levels of IL17 presenting in breast cancer patients correlate with lower survival rates of the patient [32].

## 3. Polarization States of Neutrophils in Cancer

Neutrophils respond differently to different stimuli [30]. Various stimuli in the tumor microenvironment result in the activation of neutrophils to different phenotypes as anti-tumor and pro-tumor. Similarly, with the classification of tumor-associated macrophage in the tumor microenvironment (M1 for anti-tumor microphage, M2 for pro-tumor macrophage), the neutrophils are classified into two polarization states, which are N1 (anti-tumor neutrophil) and N2 (pro-tumor neutrophil) [7]. This N1 and N2 concept were first proposed by Fridlender Z.G. et al. in 2009 [14]. According to previous studies, after exposure to regulatory factors such as G-CSF [31] or transforming growth factor β (TGF-β) [7,14], neutrophils transform to the N2 phenotype. N2 neutrophils are characterized as a higher expression of pro-tumor factors to induce the immunosuppression in the tumor microenvironment, including CCL2, CCL5, neutrophil elastase (NE), and cathepsin G (CG), with a higher expression of arginase [7,14]. Blockade of TGF-β signaling or type I IFNs treatment results in neutrophils with a hyper-segmented nucleus, and they are more cytotoxic (N1) to the tumor cells [14]. N1 neutrophils have elevated expressions of immuno-activating chemokines and cytokines including TNF (tumor necrosis factor) α, ICAM-1, and FAS [14]. The functions of neutrophils in the tumor microenvironment seem distinct. However, as of now, unlike M1 and M2, there is no suitable marker to indicate the N1 and N2 neutrophils in the tumor.

## 4. Functions of Neutrophils in the Tumor Microenvironment

### 4.1. The Pro-Cancer Role of Neutrophils

Tumor-associated neutrophils are generally considered a pro-tumor factor in multiple tumor types, including breast cancer [7,11]. Using over 5000 cases of 25 different cancer types, Gentles et al. indicated that higher polymorpho-nuclear cell (PMN, including neutrophils) infiltration would lead to the lowest overall survival for those cancer patients compared to other leukocytes [33]. Additionally, the higher neutrophil to lymphocyte ratio (NLR) indicates a worse prognosis for those patients [34,35,41,42,43,44,45,46]. There are also studies regarding neutrophils establishing a pre-metastatic niche for the malignant tumor cells [47]. These studies indicate the overall pro-tumor functions of neutrophils in multiple cancer types. We have discussed the clinical relevance of NLR in Section 5.1.

Based on the published reports, the neutrophils play pro-tumor roles through multiple mechanisms introduced below.

#### 4.1.1. Neutrophil Released Reactive Oxygen Species

As mentioned before, neutrophils are the most abundant leukocytes and are recruited to the infected site during an immune response [9]. Once the pathogen invades the host tissue and starts to replicate, the resident macrophage will respond to the pathogen with phagocytosis and secrete factors including CXCR2 ligands, which promote mobilization of activated neutrophils from bone marrow to the infected tissue [48,49]. The multiple receptors on the neutrophil surface, such as CD14, enable neutrophils to recognize and eliminate the pathogens [50]. One of the killing mechanisms of neutrophils to the pathogens includes phagocytosis. Neutrophils consist of a significant part of the phagocyte system [50]. The phagocytes can sense and engulf the pathogens by forming phagosome and later fusing with a lysosome [51]. The enzymes in the neutrophils’ granules, for instance, NADPH oxidase, enable the changes of pH in phagolysosome [52] and release the reactive oxygen species (ROS) through the respiratory burst [53]. The production of ROS in phagolysosome is for killing the pathogens [53]. However, the released ROS by neutrophils can result in DNA base damages [54], as well as mutations [55], which are essential for cancer initiation, cell proliferation, cancer-favored inflammation, immune suppression [56], and EMT in multiple cancer types, including breast cancer [57].

Tumor cells are usually subjected to high ROS levels in the tumor microenvironment [57]. Moreover, ROS usually play a pro-tumor role during progression. For instance, in breast cancer, multinucleated cells produce ROS to stabilize HIF-1α, which promote increased production of VEGF (Vascular endothelial growth factor) and MIF (Macrophage migration inhibition factor), which facilitate cancer progression and chemotherapy resistance [58]. The neutrophil released ROS can also result in epithelial damage and cancer favored inflammation [7]. ROS such as hydrogen peroxide can also function as messengers in cell signaling, which can also regulate cell signaling pathways such as the MAPK/Erk1/2pathway, the PI 3K/Akt pathway, and the IKK/NF-κB pathway in cancer [57]. CD8^+^ T cells after exposure to MDSCs produced ROS results in antigen-specific tolerance [59]. However, hydrogen peroxide production by neutrophil is also considered one of the neutrophil killing mechanisms to the tumor cells [60], so the progression of cancer requires the delicate homeostasis of ROS levels in the tumor microenvironment. The release of ROS by neutrophil can be considered a potential therapeutic target for cancer patients.

#### 4.1.2. Pro-Tumor Neutrophil-Secreted Cytokines and Chemokines

Other than ROS, neutrophils also release various cytokines and chemokines into the tumor microenvironment [61]. The production of these cytokines and chemokines differ according to different stimuli [30,62]. In the tumor microenvironment, neutrophils tend to secrete pro-tumor factors like TGF-β to educate themselves and other cell types to a pro-cancer phenotype [11,14,63]. However, in other cases like host defense, the neutrophils may behave differently compared to the tumor microenvironment. They react quickly in acute inflammation, and one of their significant roles in acute inflammation is phagocytosis [64].

Table 1 and Table 2 summarizes the cytokines and chemokines produced by neutrophils and their roles in the tumor microenvironment. Based on these studies, the dominant role of neutrophil secreted factors is pro-tumor. For example, in breast cancer, when co-cultured with human breast cancer cell lines, the neutrophils released oncostatin M, (OSM, a member of interleukin-6 (IL-6) superfamily) which promoted tumor progression by facilitating angiogenesis and metastasis through the induction of VEGF expression and increases on cancer cell invasive potential [65].

Additionally, researchers found that breast cancer metastasis required the secretion of TGF-β [80]. Tumor-associated myeloid cells expressed TGF-β, and specific deletion of *Tgfbr2* in tumor-associated myeloid cells inhibited cancer metastasis, which indicates that the myeloid-specific TGF-β signaling is a vital part of cancer metastasis [80]. Tumor-associated neutrophils can also release other pro-inflammatory cytokines into the tumor such as IL17. As described before, IL17 can upregulate CXCR2 ligand expression to facilitate neutrophils mobilization, which indicates a feedforward loop in this gastric cancer model [39]. In addition, IL17 itself can play a pro-tumor role through mechanisms such as induction of the cancer stem cell feature in pancreatic intraepithelial neoplasia cells [81]. Additionally, neutrophils release multiple chemokines into the tumor microenvironment, including CXC and CC chemokines [68,70,71,77,78]. The mobility of the neutrophil to the tumor site requires interactions between CXC chemokines in circulation and CXC receptors on the neutrophil membrane [21]. Higher levels of CXCR2 ligands including CXCL8 may result in higher numbers of recruited neutrophils on the tumor sites [82]. Therefore, the release of CXCL8 in head and neck cancer by neutrophils may suggest a feedforward loop for neutrophil recruitment [68]. In addition, in multiple cancer cases, it has been reported that neutrophils secreted a significant amount of CC ligands [71,77], and the higher levels of CC ligands correlate with lower survival rates for cancer patients [71,78]. The CC ligands are known chemoattractants for immune cells such as monocyte and regulatory T cells [83].

Apart from neutrophils, cells such as tumor cells [24], Th17 cells [40], γβ T cells [84], B cells [85], lymphocytes, and macrophages [13] present in the tumor microenvironment secrete regulatory factors to facilitate cancer progression. As discussed previously, the proliferation and maturation of neutrophils in bone marrow requires cytokines and chemokines such as G-CSF [86], CXCR2 chemokines, and IL17. Multiple cell types in the tumor microenvironment contribute to the pool of G-CSF, CXCR2 ligands, and IL17. In the tumor microenvironment, the primary source of G-CSF includes cancer cells [87], fibroblasts [88], macrophages, and lymphocytes [89] while the major contributors of IL17 include Th17 cells [90] and γβ T cells [91].

Factors secreted by neutrophils can educate other immune cells to a pro-tumor type. For instance, OSM is found to regulate macrophage polarization to a pro-tumor phenotype (M2 type) in the tumor microenvironment, and this regulation is via mTOR signaling complex 2 (mTORC2) [92]. Neutrophils also release TGF-β into the tumor microenvironment, which promotes the macrophages’ differentiation into M2 type macrophages [63]. Other than interactions with macrophages, neutrophils can interact with T cells in the tumor microenvironment [84], which can promote cancer metastasis. For example, in a breast cancer mouse model, IL17 producing T cells upregulate the levels of G-CSF, which results in the expansion of neutrophils and alters the neutrophil phenotype. The altered neutrophils then produce nitric oxide synthase (iNOS) to suppress the CD8 T cells’ anti-tumor functions in the tumor microenvironment, which results in higher metastasis of cancer cells [84].

#### 4.1.3. Neutrophil Released Enzymes

Four types of granules are present in neutrophils, the primary (azurophil), secondary, and tertiary granules, as well as secretory vesicles [93]. These granules consist of various proteases. By far, the most well studied proteases in cancer include CG, NE, and matrix metalloprotease 9 (MMP-9). They are all derived from neutrophil granules [93]. Various reports indicate that they play a pro-metastasis role through mechanisms including EMT, and extracellular matrix (ECM) remodeling [94]. For instance, NE and CG were found to degrade thrombospondin 1 in the pre-metastatic tumor microenvironment to promote cancer progression [95].

CG is a serine protease that resides in neutrophil primary granules. CG is pre-synthesized in promyelocytes in bone marrow and then stored in neutrophil primary granules as active proteases. The high isoelectric points for CG (12) cause them to be easily caught in negatively charged traps such as neutrophil extracellular traps (NETs) [96]. In breast cancer, CG facilitated the E-cadherin-dependent aggregation of mammary carcinoma cells, MCF-7 [97], and it was through insulin-like growth factor-1 signaling [98]. Inhibition of CG resulted in less osteolysis in breast cancer, which indicated CG as a potential therapeutic target [99].

NE is also known as a serine protease. Neutrophils mostly contributed NE. Similar to CG, NE is pre-synthesized in promyelocytes and stored in neutrophil granules in an active form. The high isoelectric points for NE (larger than 9) also cause them to be easily trapped in negative charged NET [96]. NE is found to initiate and upregulate the cancer-related signaling such as EGFR/MEK/ERK signaling [100], and phosphatidylinositol 3-kinase (PI3K) signaling [101]. Interactions between NE and signaling results in higher levels of pro-cancer factors such as TGF-β [102].

NE significantly promotes cancer cell proliferation, metastasis, and therapy resistance [103,104]. Cancer cells can uptake NE through neuropilin-1 if they lack endogenous NE expression [105]. Various studies showed inhibition of NE results in the suppression of tumor progression in multiple cancer types, including breast and prostate cancer [104,106]. Breast cancer patients with higher levels of NE correlated with lower survival rates, which indicates NE as an independent prognostic marker [107]. Additionally, the increased expression level of NE is suggested to be a therapeutic target for colorectal cancer [108].

NE and CG both promote lung metastasis by degrading anti-cancer protein Thrombospondin 1 (Tsp1) [95]. Both CG and NE are also involved in ECM remodeling in the tumor microenvironment [94]. Moreover, ECM remodeling is very crucial for cancer metastasis. Other than NE and CG, neutrophil released matrix metalloproteases (MMP) such as MMP-8 and MMP-9 are also found to be involved in ECM remodeling to facilitate cancer progression [94,109].

MMP is defined as a cluster of enzymes whose catalytic abilities require the involvement of zinc [110]. MMP-9 is stored in neutrophil tertiary granules [111]. The release of MMP-9 is delicately regulated by various cytokines and growth factors, including the TNF, TGF-β, and the VEGF [111,112,113]. After release from the neutrophil granules, MMP-9 plays a pro-tumor role through mechanisms such as remodeling of ECM by degradation of extracellular proteins (such as type IV collagen) [110], membrane cleavage [114], or activating pro-tumor factors including TGF-β [115].

Compared with normal tissues, breast cancer tissues have higher expression levels of MMP-9, which suggests it is associated with breast cancer development and tumor progression [116]. In basal-like triple negative breast cancer, MMP-9 significantly promotes breast cancer metastasis and angiogenesis [117]. Silencing MMP-9 expression results in suppression of malignancy [117]. Higher MMP-9 levels also indicate the more severe malignancies and shorter survival time. Studies indicate that higher levels of MMP-9 correlate with higher metastasis in breast cancer patients [118]. Positive stromal MMP-9 expression also predicts poor survival in hormone-responsive small mammary tumors [119]. All these results indicate MMP-9 as a potential biomarker for breast cancer patients.

#### 4.1.4. NET

Historically, it was thought that neutrophil killing mechanisms included phagocytosis and secretion of killing factors such as MMPs, CG, and NE. However, in 2004, Brinkmann et al. discovered NET as another killing mechanism [120]. This mechanism is named NETosis. NETosis is a unique form of cell death that is characterized by the release of de-condensed chromatin and granular contents to the extracellular space. NETosis usually requires stimulation to neutrophils and the generation of ROS by NADPH oxidase [121]. Initial studies report the neutrophils after being activated by stimuli such as CXCL8 or lipopolysaccharide (LPS) will produce the fragile and fiber-like net by ejecting nuclear chromatin attached with proteases (such as NE, CG, MMP-9, myeloperoxidase) to entangle and eliminate the pathogens. This process requires the rupture of the cytoplasmic membrane [122]. However, there are also reports regarding neutrophils forming NET through the release of mitochondria DNA. This process does not require the lytic death of neutrophils [122,123,124]. Additionally, NET plays a regulatory role in multiple diseases by activating dendritic and T cells [125].

Neutrophils are also activated and form NET in the tumor microenvironment. Based on previous studies, NET plays a pro-tumor role during tumor progression [126,127]. There is evidence that indicates the NET directly functions on tumors cells, which enhances their proliferation through proteases such as NE on the NET, or through activating signaling pathways such as the NF-κB [128]. Additionally, in a lung cancer model, NET trapped the circulating lung carcinoma cells and promoted tumor cell metastasis [129]. Further studies demonstrate the capture of cancer cells by neutrophils is through β1-integrin expressions on both cancer cells and NETs [130].

When compared to healthy controls, the levels of NET increased in cancer patients’ plasma (lung cancer, pancreatic adenocarcinoma, and bladder cancer) [131]. Furthermore, in Ewing sarcoma, patients with higher levels of NET have metastasis and early relapse after intensive chemotherapy treatment [125]. Similarly, according to these findings, the levels of NETs in colorectal cancer patients are also significantly higher than healthy controls. Adverse patient outcomes are associated with increased preoperative NETs production [132]. These results indicate that NETs could be considered a potential prognostic marker and therapeutic target.

In breast cancer, LPS-activated neutrophils awaken the dormant breast cancer cells by producing NET [133]. The produced NET remodels laminin through the MMP-9 and NE proteases on the NET. The remodeled laminin further activates integrin α3β1 signaling to awaken the breast cancer cells. Inhibiting the formation of NET by DNase I digestion or by inhibition of protein arginine deiminase 4 prevents the activation of dormant cancer cells [133]. Additionally, metastatic breast cancer cells are also able to activate neutrophils and promote the formation of NET in the absence of infection [134]. The activation of neutrophils by cancer cells is through the secretion of G-CSF. Blocking the formation of NET by DNase I showed the prevention of lung metastasis in mice [134].

#### 4.1.5. Neutrophil and Therapy Resistance

Chemotherapy as the first line defense is most commonly used for cancer patients. However, one of the major challenges regarding cancer treatments includes therapeutic resistance. Currently, researchers found the tumor microenvironment is closely linked with therapy resistance. The changes in the tumor microenvironment include polarization of immune cells to a pro-tumor type as well as secretion of cytokines and proteases that promotes angiogenesis and metastasis [135].

Neutrophils are a significant component in the tumor microenvironment and are found to play a pivotal role in chemotherapy resistance. The potential prognostic marker NLR is a useful marker for resistance to chemotherapy. The higher NLR indicates higher resistance when patients receive chemotherapy drugs, which indicates the lower survival rates for cancer patients [34,35,136]. Targeting neutrophils in pancreatic ductal adenocarcinoma enhances the therapeutic response to chemotherapy drugs [137]. Neutrophils can release pro-angiogenic factors such as MMP-9, MMP-8, and CXCL8 to promote resistance to sunitinib in renal cell carcinoma patients [138]. Sunitinib is a common chemotherapy drug for multiple cancer types. Additionally, higher levels of NE in distant metastatic breast cancer patients is associated with a poor response to tamoxifen [139]. Neutrophil released TGF-β can also be involved in the EMT process, which promotes tumor cells establishing resistance to gemcitabine [140]. Other than facilitating cancer cell resistance to chemotherapy drugs, neutrophils also facilitate tumor cell resistance to anti-angiogenesis therapy [141]. For instance, IL17 promotes resistance to VEGF inhibition therapy by positively recruiting neutrophils into the tumor microenvironment [142].

### 4.2. The Anti-Cancer Role of Neutrophils

**Figure 1:**
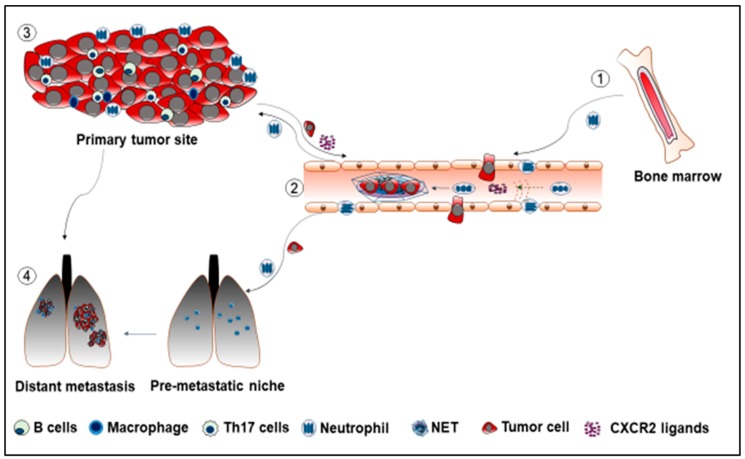
① Neutrophils mobilize from bone marrow, enter the circulatory system, and move using a chemotactic way to the primary tumor sites. ② Neutrophils promote cancer metastasis through the formation of NET. The NET traps the dormant tumor cells, which facilitates the establishment of the secondary tumor sites. ③ Cells in the tumor microenvironment release pro-tumor factors such as CXCR2 ligands into the circulatory system to recruit neutrophils to the tumor sites. ④ Neutrophils arrive in the pre-metastatic lung to establish the pre-metastatic niche for tumor cells.

The majority of cancer studies report that neutrophils are playing a pro-tumor progression role (Figure 1). Nevertheless, some published data also indicates that neutrophils play an anti-tumor role in certain cases. For instance, neutrophils after physical contact with cancer cells can secret H_2_O_2_, which results in tumor cell death via Ca^2+^ influx through the TRPM2 Ca^2+^ channel [143]. Furthermore, through physical contact, neutrophils isolated from healthy donors specifically possess a tumor suppression ability mediated through Fas ligand /Fas interaction [144]. The Met and its ligand, the hepatocyte growth factor (HGF), also caused the release of nitric oxide to eliminate the tumor cells in anti-tumor neutrophils [145]. Preclinical studies to educate pro-tumor neutrophils to an anti-tumor type suggest their utility. The combination of poly I:C and inactivated Sendai virus particles (hemagglutinating virus of Japan envelope; HVJ-E) increased the FAS^+^ neutrophil infiltration in the tumor. Concurrently, FAS^+^ neutrophils enhanced cytotoxic T lymphocyte activity towards B16-F10 melanoma cells [146].

The majority of neutrophil-released proteases play a pro-tumor role such as NE and CG. Furthermore, neutrophils also release MMP-8 (Collagenase-2) in cancer cases. MMP-8 modulates neutrophil mobilization by generating chemotactic Pro-Gly-Pro (PGP) tripeptide [147]. However, the role of MMP-8 in tumor progression is controversial. Some reports indicate the anti-tumor role of MMP-8. For instance, in syngeneic melanoma and the lung carcinoma mouse models, MMP-8 prevented metastasis formation by regulating tumor cell adhesion and invasion. Cells with MMP-8 overexpression enhanced adhesion to type I collagen and laminin-1, and knock-off of MMP-8 in mice resulted in increased metastasis [148].

In breast cancer patients, expression of MMP-8 correlates with lower lymph node metastasis, which indicates MMP-8 as a potential prognostic marker for breast cancer patients [148]. Although the expression of MMP-8 is detrimental to breast cancer cells, MMP-8 upregulates pro-tumor cytokines, IL-6 and IL-8, in a self-reinforcing loop manner [149]. In colorectal cancer patients’ serum, higher levels of MMP-8 indicate an adverse outcome for the patient [150].

## 5. The Clinical Significance of Neutrophils

Based on recent findings, neutrophils in the tumor microenvironment usually play a pro-tumor role through the formation of NET, the release of ROS, the secretion of pro-tumor cytokines and chemokines, and the promotion of immunosuppression. Neutrophils emerged as the least favorable cell populations regarding cancer patients’ survival, which indicates significance for patients’ prognosis [33].

### 5.1. Neutrophils as A Potential Biomarker for Cancer Patients

Higher infiltration of neutrophils or NLR in cancer patients correlates with poor clinical outcomes in multiple cancers [33,34,35,42,43,45,46,151,152]. All these findings indicate that neutrophils could be considered a potential prognostic marker for cancer patients. When a significantly high number of infiltrated neutrophils are present in tumors compared to normal tissues, neutrophils may serve as a diagnostic indicator [108,153]. Other than the neutrophil itself, the neutrophil releasing factors NE and OSM could also be considered a prognostic and diagnostic marker for multiple cancer types, including breast cancer [107,108,154]. The detection of neutrophil frequency or NLR, or neutrophil-releasing factors in patients’ serum is easy, inexpensive, and applicable [152]. However, to make the prognostic or diagnostic results more accurate, the results may still require optimization by a combination of other cancer-related factors.

### 5.2. Targeting Neutrophils in Cancer: The Therapeutic Plan

As the most abundant leukocyte in the human circulation system, the role of neutrophils in tumor progression is pivotal. Additionally, neutrophils always play a pro-tumor role in the tumor microenvironment. Depletion of neutrophils prevented cancer progression in various mouse models [155]. Targeting neutrophils or neutrophil-releasing factors could be regarded as a promising therapeutic plan for cancer patients.

Based on previous research, there are mainly four ways to target neutrophils: prevention of neutrophil expansion in the bone marrow, inhibition of neutrophil recruitment to the tumor or circulation system, education of pro-tumor neutrophils to an anti-tumor phenotype, and targeting neutrophil-releasing pro-tumor factors (for instance, neutrophil released cytokines and proteases).

Clinically, one of the most applicable ways of targeting neutrophils is through the inhibition of CXCR2, which is the positive regulator of neutrophil mobilization. For instance, a CXCR2 antagonist, AZD5069, effectively reduced absolute neutrophil counts in bronchiectasis patients [156]. The AZD5069 anti-cancer effect and whether it could be included as a therapeutic plan for cancer patients is still under investigation. Additionally, there are studies regarding targeting neutrophils-releasing NE in cancer patients. The application of the NE inhibitor to cancer patients mostly focuses on alleviating the side effects of the therapeutic cancer plan. For example, after receiving the NE inhibitor, sivelestat sodium hydrate, patients with thoracic esophagus carcinoma showed an improved systemic inflammatory response [157]. Elimination of NET by DNase I digestion is also an applicable and easy method [134]. However, although several clinical trials are ongoing, no result has come out regarding targeting NET in cancer patients. Additional preclinical and clinical studies are needed to better understand the therapeutic effects of targeting neutrophils in cancer patients.

## 6. Conclusions

Currently, more attention is being directed toward tumor-associated neutrophils and their functions in the tumor microenvironment. In recent years, researchers have generated data regarding neutrophil’s extended survival time, NETosis, and N1 and N2 polarization states. However, more data is still needed to delineate the neutrophil facilitated tumor progression, and it may take time to translate these research results to clinical use for cancer patients. Nevertheless, studies on neutrophils shed light on understanding the tumor microenvironment, which promotes more research in order to find a cure for cancer patients.

## Figures and Tables

**Table 1:** Neutrophil produced cytokines and chemokines in human cancer.

Cancer Type	Cytokine/Chemokine	Stimulator	Target Cell	Effect	Reference
Breast cancer	Oncostatin M	GM-CSF signals. (cell-cell contact needed.)	Breast cancer cells	Induces vascular endothelial growth factor expression, cell detachment, and increases invasive potential.	[65]
Adenocarcinoma of the bronchioloalveolar carcinoma (BAC) subtype	HGF	GM-CSF TNFα	BCA tumor cells	Promotes migration of tumor cells and is also positively associated with poorer outcome in BAC patients. An independent predictor of clinical outcome in multivariate analysis.	[66]
Pancreatic cancer	TGF-β	Not shown	Not shown	Results in overproduction of collagens in pancreatic cancer, which ends in desmoplastic reaction.	[67]
Head and neck cancer	CCL4 and CXCL8	P38-MAPK	Not shown	Pro-tumor chemokine	[68]
Gastric cancer	IL17	TAM derived IL-6 and IL-23	Neutrophils	Pro-inflammatory IL17 is a critical mediator of the recruitment of neutrophils into the invasive margin by CXC chemokines.	[39]
Lung Adenocarcinoma	BV8 (Prok2)	G-CSF GM-CSF	Neutrophil	Promotes neutrophil chemotaxis.	[69]
Thyroid cancer	CXCL8, VEGF-A, and TNF-α	Unknown	Not shown	Pro-inflammatory and angiogenic mediators.	[70]
Hepatocellular carcinoma	CCL2, CCL3	Unknown	Immune cells in the tumor microenvironment	The survival rate of the CCL2 high group was significantly lower than other patients. Host immune suppression.	[71]
Oral cavity cancer	VEGF, IL-18	Unknown	Not shown	May promote neoangiogenesis and metastatic cancer in the early stage of oral cavity cancer.	[72]
Bladder cancer	CCL2, CCL3, CCL4, G-CSF, and IL-6	Unknown	Not shown	Pro-inflammatory cytokine and chemokines.	[73]

**Table 2:** Neutrophil produced cytokines and chemokines in the mouse model.

Cancer Type	Cytokine/Chemokine	Stimulator	Target Cell	Effect	Reference
Melanoma	VEGF	Not shown	Not shown	Promotes angiogenesis, tumor metastasis.	[74]
Colitis-associated cancer	(IL)-1β (expression also seen in human neutrophils)	Not shown	Intestinal mononuclear phagocytes upregulate IL 6.	Induces tumor formation.	[75]
Colon cancer	IL10	Not shown	Regulates STAT3 activation to upregulate DNMT3b to silence tumor suppressor IRF8 in colonic epithelial cells.	Facilitates colon cancer initiation.	[76]
Mesothelioma and lung carcinoma	CCL17 (expression also seen in human neutrophils)	Not shown	Regulatory T-cells	Facilitates the recruitment of regulatory T cells, which results in an immuno-suppressive nature.	[77]
Breast cancer (Gr-1+CD11b+ immature myeloid cells)	CCL9	TGF-β signaling	Myeloid cells	Promotes tumor cell survival in the pre-metastatic organ.	[78]
Breast cancer	Prokineticin 2 (Prok2)	Not shown	Tumor cells	Enhances tumor cell proliferation.	[79]

## References

[1] Siegel R.L., Miller K.D., Jemal A. (2018). Cancer statistics, 2018. CA Cancer J. Clin..

[2] Hsu P.L., Jou J., Tsai S.J. (2019). TYRO3: A potential therapeutic target in cancer. Exp. Biol. Med..

[3] Dutcher J.P., Novik Y., O’Boyle K., Marcoullis G., Secco C., Wiernik P.H. (2000). 20th-century advances in drug therapy in oncology—Part. II. J. Clin. Pharmacol..

[4] Jo Y., Choi N., Kim K., Koo H.J., Choi J., Kim H.N. (2018). Chemoresistance of Cancer Cells: Requirements of Tumor Microenvironment-mimicking In Vitro Models in Anti-Cancer Drug Development. Theranostics.

[5] Zahreddine H., Borden K.L. (2013). Mechanisms and insights into drug resistance in cancer. Front. Pharmacol..

[6] Peinado H., Zhang H., Matei I.R., Costa-Silva B., Hoshino A., Rodrigues G., Psaila B., Kaplan R.N., Bromberg J.F., Kang Y. (2017). Pre-metastatic niches: Organ-specific homes for metastases. Nat. Rev. Cancer.

[7] Coffelt S.B., Wellenstein M.D., de Visser K.E. (2016). Neutrophils in cancer: Neutral no more. Nat. Rev. Cancer.

[8] Selders G.S., Fetz A.E., Radic M.Z., Bowlin G.L. (2017). An overview of the role of neutrophils in innate immunity, inflammation and host-biomaterial integration. Regen. Biomater..

[9] Kruger P., Saffarzadeh M., Weber A.N., Rieber N., Radsak M., von Bernuth H., Benarafa C., Roos D., Skokowa J., Hartl D. (2015). Neutrophils: Between host defense, immune modulation, and tissue injury. PLoS Pathog..

[10] Arati Khanna-Gupta N.B., Ronald Hoffman E.J.B., Silberstein L.E., Heslop H.E., Weitz J.I., Anastasi J., Salama M.E., Abutalib S.A. (2018). Hematology (Seventh Edition).

[11] Shaul M.E., Fridlender Z.G. (2017). Neutrophils as active regulators of the immune system in the tumor microenvironment. J. Leukoc. Biol..

[12] Pillay J., den Braber I., Vrisekoop N., Kwast L.M., de Boer R.J., Borghans J.A., Tesselaar K., Koenderman L. (2010). In vivo labeling with 2H_2_O reveals a human neutrophil lifespan of 5.4 days. Blood.

[13] Akgul C., Moulding D.A., Edwards S.W. (2001). Molecular control of neutrophil apoptosis. FEBS Lett..

[14] Fridlender Z.G., Sun J., Kim S., Kapoor V., Cheng G., Ling L., Worthen G.S., Albelda S.M. (2009). Polarization of tumor-associated neutrophil phenotype by TGF-β: “N1” versus “N2” TAN. Cancer Cell.

[15] Triner D., Devenport S.N., Ramakrishnan S.K., Ma X., Frieler R.A., Greenson J.K., Inohara N., Nunez G., Colacino J.A., Mortensen R.M. (2018). Neutrophils Restrict Tumor-Associated Microbiota to Reduce Growth and Invasion of Colon Tumors in Mice. Gastroenterology.

[16] Gabrilovich D.I., Nagaraj S. (2009). Myeloid-derived suppressor cells as regulators of the immune system. Nat. Rev. Immunol..

[17] Furze R.C., Rankin S.M. (2008). Neutrophil mobilization and clearance in the bone marrow. Immunology.

[18] Elghetany M.T. (2002). Surface antigen changes during normal neutrophilic development: A critical review. Blood Cell. Mol. Dis..

[19] Martin C., Burdon P.C., Bridger G., Gutierrez-Ramos J.C., Williams T.J., Rankin S.M. (2003). Chemokines acting via CXCR2 and CXCR4 control the release of neutrophils from the bone marrow and their return following senescence. Immunity.

[20] Chow M.T., Luster A.D. (2014). Chemokines in cancer. Cancer Immunol. Res..

[21] Eash K.J., Greenbaum A.M., Gopalan P.K., Link D.C. (2010). CXCR2 and CXCR4 antagonistically regulate neutrophil trafficking from murine bone marrow. J. Clin. Investig..

[22] Hong C.W. (2017). Current Understanding in Neutrophil Differentiation and Heterogeneity. Immune Netw..

[23] Belperio J.A., Keane M.P., Burdick M.D., Londhe V., Xue Y.Y., Li K., Phillips R.J., Strieter R.M. (2002). Critical role for CXCR2 and CXCR2 ligands during the pathogenesis of ventilator-induced lung injury. J. Clin. Investig..

[24] Sharma B., Nawandar D.M., Nannuru K.C., Varney M.L., Singh R.K. (2013). Targeting CXCR2 enhances chemotherapeutic response, inhibits mammary tumor growth, angiogenesis, and lung metastasis. Mol. Cancer Ther..

[25] Fridlender Z.G., Albelda S.M. (2012). Tumor-associated neutrophils: Friend or foe?. Carcinogenesis.

[26] Wang N., Liu W., Zheng Y., Wang S., Yang B., Li M., Song J., Zhang F., Zhang X., Wang Q. (2018). CXCL1 derived from tumor-associated macrophages promotes breast cancer metastasis via activating NF-kappaB/SOX4 signaling. Cell Death Dis..

[27] Chan T.S., Hsu C.C., Pai V.C., Liao W.Y., Huang S.S., Tan K.T., Yen C.J., Hsu S.C., Chen W.Y., Shan Y.S. (2016). Metronomic chemotherapy prevents therapy-induced stromal activation and induction of tumor-initiating cells. J. Exp. Med..

[28] Wu Y., Wang S., Farooq S.M., Castelvetere M.P., Hou Y., Gao J.L., Navarro J.V., Oupicky D., Sun F., Li C. (2012). A chemokine receptor CXCR2 macromolecular complex regulates neutrophil functions in inflammatory diseases. J. Biol. Chem..

[29] Girbl T., Lenn T., Perez L., Rolas L., Barkaway A., Thiriot A., Del Fresno C., Lynam E., Hub E., Thelen M. (2018). Distinct Compartmentalization of the Chemokines CXCL1 and CXCL2 and the Atypical Receptor ACKR1 Determine Discrete Stages of Neutrophil Diapedesis. Immunity.

[30] Scapini P., Lapinet-Vera J.A., Gasperini S., Calzetti F., Bazzoni F., Cassatella M.A. (2000). The neutrophil as a cellular source of chemokines. Immunol. Rev..

[31] Casbon A.J., Reynaud D., Park C., Khuc E., Gan D.D., Schepers K., Passegue E., Werb Z. (2015). Invasive breast cancer reprograms early myeloid differentiation in the bone marrow to generate immunosuppressive neutrophils. Proc. Natl Acad. Sci. USA.

[32] Yang B., Kang H., Fung A., Zhao H., Wang T., Ma D. (2014). The role of interleukin 17 in tumour proliferation, angiogenesis, and metastasis. Mediators Inflamm..

[33] Gentles A.J., Newman A.M., Liu C.L., Bratman S.V., Feng W., Kim D., Nair V.S., Xu Y., Khuong A., Hoang C.D. (2015). The prognostic landscape of genes and infiltrating immune cells across human cancers. Nat. Med..

[34] Lorente D., Mateo J., Templeton A.J., Zafeiriou Z., Bianchini D., Ferraldeschi R., Bahl A., Shen L., Su Z., Sartor O. (2015). Baseline neutrophil-lymphocyte ratio (NLR) is associated with survival and response to treatment with second-line chemotherapy for advanced prostate cancer independent of baseline steroid use. Ann. Oncol..

[35] Gonda K., Shibata M., Sato Y., Washio M., Takeshita H., Shigeta H., Ogura M., Oka S., Sakuramoto S. (2017). Elevated neutrophil-to-lymphocyte ratio is associated with nutritional impairment, immune suppression, resistance to S-1 plus cisplatin, and poor prognosis in patients with stage IV gastric cancer. Mol. Clin. Oncol..

[36] Akbay E.A., Koyama S., Liu Y., Dries R., Bufe L.E., Silkes M., Alam M.M., Magee D.M., Jones R., Jinushi M. (2017). Interleukin-17A Promotes Lung Tumor Progression through Neutrophil Attraction to Tumor Sites and Mediating Resistance to PD-1 Blockade. J. Thorac. Oncol..

[37] Hirai Y., Iyoda M., Shibata T., Kuno Y., Kawaguchi M., Hizawa N., Matsumoto K., Wada Y., Kokubu F., Akizawa T. (2012). IL-17A stimulates granulocyte colony-stimulating factor production via ERK1/2 but not p38 or JNK in human renal proximal tubular epithelial cells. Am. J. Physiol. Renal. Physiol..

[38] Hata K., Andoh A., Shimada M., Fujino S., Bamba S., Araki Y., Okuno T., Fujiyama Y., Bamba T. (2002). IL-17 stimulates inflammatory responses via NF-kappaB and MAP kinase pathways in human colonic myofibroblasts. Am. J. Physiol. Gastrointest. Liver Physiol..

[39] Li T.J., Jiang Y.M., Hu Y.F., Huang L., Yu J., Zhao L.Y., Deng H.J., Mou T.Y., Liu H., Yang Y. (2017). Interleukin-17-Producing Neutrophils Link Inflammatory Stimuli to Disease Progression by Promoting Angiogenesis in Gastric Cancer. Clin. Cancer Res..

[40] Novitskiy S.V., Pickup M.W., Gorska A.E., Owens P., Chytil A., Aakre M., Wu H., Shyr Y., Moses H.L. (2011). TGF-β receptor II loss promotes mammary carcinoma progression by Th17 dependent mechanisms. Cancer Discov..

[41] Ethier J.L., Desautels D., Templeton A., Shah P.S., Amir E. (2017). Prognostic role of neutrophil-to-lymphocyte ratio in breast cancer: A systematic review and meta-analysis. Breast Cancer Res..

[42] Suzuki R., Takagi T., Hikichi T., Konno N., Sugimoto M., Watanabe K.O., Nakamura J., Waragai Y., Kikuchi H., Takasumi M. (2016). Derived neutrophil/lymphocyte ratio predicts gemcitabine therapy outcome in unresectable pancreatic cancer. Oncol. Lett..

[43] Mimica X., Acevedo F., Oddo D., Ibanez C., Medina L., Kalergis A., Camus M., Sanchez C. (2016). [Neutrophil/lymphocyte ratio in complete blood count as a mortality predictor in breast cancer]. Rev. Med. Chil..

[44] Graziano V., Grassadonia A., Iezzi L., Vici P., Pizzuti L., Barba M., Quinzii A., Camplese A., Di Marino P., Peri M. (2019). Combination of peripheral neutrophil-to-lymphocyte ratio and platelet-to-lymphocyte ratio is predictive of pathological complete response after neoadjuvant chemotherapy in breast cancer patients. Breast.

[45] Doi H., Nakamatsu K., Anami S., Fukuda K., Inada M., Tatebe H., Ishikawa K., Kanamori S., Monzen H., Nishimura Y. (2019). Neutrophil-to-Lymphocyte Ratio Predicts Survival After Whole-brain Radiotherapy in Non-small Cell Lung Cancer. In Vivo.

[46] Zhao L., Li T., Yang Y., Zhang Y., Li W., Han L., Shang Y., Lin H., Ren X., Gao Q. (2019). Clinical value of neutrophil-to-lymphocyte ratio as a predictor of prognosis of RetroNectin((R))-activated cytokine-induced killer cell therapy in advanced non-small-cell lung cancer. Immunotherapy.

[47] Wculek S.K., Malanchi I. (2015). Neutrophils support lung colonization of metastasis-initiating breast cancer cells. Nature.

[48] De Oliveira S., Rosowski E.E., Huttenlocher A. (2016). Neutrophil migration in infection and wound repair: Going forward in reverse. Nat. Rev. Immunol..

[49] Prame Kumar K., Nicholls A.J., Wong C.H.Y. (2018). Partners in crime: Neutrophils and monocytes/macrophages in inflammation and disease. Cell Tissue Res..

[50] Dale D.C., Boxer L., Liles W.C. (2008). The phagocytes: Neutrophils and monocytes. Blood.

[51] Peyron P., Maridonneau-Parini I., Stegmann T. (2001). Fusion of human neutrophil phagosomes with lysosomes in vitro: Involvement of tyrosine kinases of the SRC family and inhibition by mycobacteria. J. Biol. Chem..

[52] Jankowski A., Scott C.C., Grinstein S. (2002). Determinants of the phagosomal pH in neutrophils. J. Biol. Chem..

[53] Winterbourn C.C., Kettle A.J., Hampton M.B. (2016). Reactive Oxygen Species and Neutrophil Function. Annu. Rev. Biochem..

[54] Cadet J., Wagner J.R. (2013). DNA base damage by reactive oxygen species, oxidizing agents, and UV radiation. Cold Spring Harb. Perspect. Biol..

[55] Cooke M.S., Evans M.D., Dizdaroglu M., Lunec J. (2003). Oxidative DNA damage: Mechanisms, mutation, and disease. FASEB J..

[56] Uribe-Querol E., Rosales C. (2015). Neutrophils in Cancer: Two Sides of the Same Coin. J. Immunol. Res..

[57] Liou G.Y., Storz P. (2010). Reactive oxygen species in cancer. Free Radic. Res..

[58] Parekh A., Das S., Parida S., Das C.K., Dutta D., Mallick S.K., Wu P.H., Kumar B.N.P., Bharti R., Dey G. (2018). Multi-nucleated cells use ROS to induce breast cancer chemo-resistance in vitro and in vivo. Oncogene.

[59] Nagaraj S., Gupta K., Pisarev V., Kinarsky L., Sherman S., Kang L., Herber D.L., Schneck J., Gabrilovich D.I. (2007). Altered recognition of antigen is a mechanism of CD8+ T cell tolerance in cancer. Nat. Med..

[60] Dallegri F., Ottonello L., Ballestrero A., Dapino P., Ferrando F., Patrone F., Sacchetti C. (1991). Tumor cell lysis by activated human neutrophils: Analysis of neutrophil-delivered oxidative attack and role of leukocyte function-associated antigen 1. Inflammation.

[61] Powell D.R., Huttenlocher A. (2016). Neutrophils in the Tumor Microenvironment. Trends Immunol..

[62] Tecchio C., Scapini P., Pizzolo G., Cassatella M.A. (2013). On the cytokines produced by human neutrophils in tumors. Semin. Cancer Biol..

[63] Zhang F., Wang H., Wang X., Jiang G., Liu H., Zhang G., Wang H., Fang R., Bu X., Cai S. (2016). TGF-β induces M2-like macrophage polarization via SNAIL-mediated suppression of a pro-inflammatory phenotype. Oncotarget.

[64] Silva M.T., Correia-Neves M. (2012). Neutrophils and macrophages: The main partners of phagocyte cell systems. Front. Immunol..

[65] Queen M.M., Ryan R.E., Holzer R.G., Keller-Peck C.R., Jorcyk C.L. (2005). Breast cancer cells stimulate neutrophils to produce oncostatin M: Potential implications for tumor progression. Cancer Res..

[66] Wislez M., Rabbe N., Marchal J., Milleron B., Crestani B., Mayaud C., Antoine M., Soler P., Cadranel J. (2003). Hepatocyte growth factor production by neutrophils infiltrating bronchioloalveolar subtype pulmonary adenocarcinoma: Role in tumor progression and death. Cancer Res..

[67] Aoyagi Y., Oda T., Kinoshita T., Nakahashi C., Hasebe T., Ohkohchi N., Ochiai A. (2004). Overexpression of TGF-β by infiltrated granulocytes correlates with the expression of collagen mRNA in pancreatic cancer. Br. J. Cancer.

[68] Dumitru C.A., Fechner M.K., Hoffmann T.K., Lang S., Brandau S. (2012). A novel p38-MAPK signaling axis modulates neutrophil biology in head and neck cancer. J. Leukoc. Biol..

[69] Zhong C., Qu X., Tan M., Meng Y.G., Ferrara N. (2009). Characterization and regulation of bv8 in human blood cells. Clin. Cancer Res..

[70] Galdiero M.R., Varricchi G., Loffredo S., Bellevicine C., Lansione T., Ferrara A.L., Iannone R., di Somma S., Borriello F., Clery E. (2018). Potential involvement of neutrophils in human thyroid cancer. PLoS ONE.

[71] Tsuda Y., Fukui H., Asai A., Fukunishi S., Miyaji K., Fujiwara S., Teramura K., Fukuda A., Higuchi K. (2012). An immunosuppressive subtype of neutrophils identified in patients with hepatocellular carcinoma. J. Clin. Biochem. Nutr..

[72] Jablonska E., Puzewska W., Grabowska Z., Jablonski J., Talarek L. (2005). VEGF, IL-18 and NO production by neutrophils and their serum levels in patients with oral cavity cancer. Cytokine.

[73] Eruslanov E., Neuberger M., Daurkin I., Perrin G.Q., Algood C., Dahm P., Rosser C., Vieweg J., Gilbert S.M., Kusmartsev S. (2012). Circulating and tumor-infiltrating myeloid cell subsets in patients with bladder cancer. Int. J. Cancer.

[74] Jablonska J., Leschner S., Westphal K., Lienenklaus S., Weiss S. (2010). Neutrophils responsive to endogenous IFN-beta regulate tumor angiogenesis and growth in a mouse tumor model. J. Clin. Investig..

[75] Wang Y., Wang K., Han G.C., Wang R.X., Xiao H., Hou C.M., Guo R.F., Dou Y., Shen B.F., Li Y. (2014). Neutrophil infiltration favors colitis-associated tumorigenesis by activating the interleukin-1 (IL-1)/IL-6 axis. Mucosal Immunol..

[76] Ibrahim M.L., Klement J.D., Lu C., Redd P.S., Xiao W., Yang D., Browning D.D., Savage N.M., Buckhaults P.J., Morse H.C. (2018). Myeloid-Derived Suppressor Cells Produce IL-10 to Elicit DNMT3b-Dependent IRF8 Silencing to Promote Colitis-Associated Colon Tumorigenesis. Cell Rep..

[77] Mishalian I., Bayuh R., Eruslanov E., Michaeli J., Levy L., Zolotarov L., Singhal S., Albelda S.M., Granot Z., Fridlender Z.G. (2014). Neutrophils recruit regulatory T-cells into tumors via secretion of CCL17—A new mechanism of impaired antitumor immunity. Int. J. Cancer.

[78] Yan H.H., Jiang J., Pang Y., Achyut B.R., Lizardo M., Liang X., Hunter K., Khanna C., Hollander C., Yang L. (2015). CCL9 Induced by TGF-β Signaling in Myeloid Cells Enhances Tumor Cell Survival in the Premetastatic Organ. Cancer Res..

[79] Sasaki S., Baba T., Muranaka H., Tanabe Y., Takahashi C., Matsugo S., Mukaida N. (2018). Involvement of Prokineticin 2-expressing Neutrophil Infiltration in 5-Fluorouracil-induced Aggravation of Breast Cancer Metastasis to Lung. Mol. Cancer Ther..

[80] Pang Y., Gara S.K., Achyut B.R., Li Z., Yan H.H., Day C.P., Weiss J.M., Trinchieri G., Morris J.C., Yang L. (2013). TGF-β signaling in myeloid cells is required for tumor metastasis. Cancer Discov..

[81] Zhang Y., Zoltan M., Riquelme E., Xu H., Sahin I., Castro-Pando S., Montiel M.F., Chang K., Jiang Z., Ling J. (2018). Immune Cell Production of Interleukin 17 Induces Stem Cell Features of Pancreatic Intraepithelial Neoplasia Cells. Gastroenterology.

[82] De Oliveira S., Reyes-Aldasoro C.C., Candel S., Renshaw S.A., Mulero V., Calado A. (2013). Cxcl8 (IL-8) mediates neutrophil recruitment and behavior in the zebrafish inflammatory response. J. Immunol..

[83] Sokol C.L., Luster A.D. (2015). The chemokine system in innate immunity. Cold Spring Harb. Perspect. Biol..

[84] Coffelt S.B., Kersten K., Doornebal C.W., Weiden J., Vrijland K., Hau C.S., Verstegen N.J.M., Ciampricotti M., Hawinkels L., Jonkers J. (2015). IL-17-producing gammadelta T cells and neutrophils conspire to promote breast cancer metastasis. Nature.

[85] Bodogai M., Moritoh K., Lee-Chang C., Hollander C.M., Sherman-Baust C.A., Wersto R.P., Araki Y., Miyoshi I., Yang L., Trinchieri G. (2015). Immunosuppressive and Prometastatic Functions of Myeloid-Derived Suppressive Cells Rely upon Education from Tumor-Associated B Cells. Cancer Res..

[86] Bottoni U., Trapasso F. (2009). The role of G-CSF in the treatment of advanced tumors. Cancer Biol. Ther..

[87] Aliper A.M., Frieden-Korovkina V.P., Buzdin A., Roumiantsev S.A., Zhavoronkov A. (2014). A role for G-CSF and GM-CSF in nonmyeloid cancers. Cancer Med..

[88] Dorsam B., Bosl T., Reiners K.S., Barnert S., Schubert R., Shatnyeva O., Zigrino P., Engert A., Hansen H.P., von Strandmann E.P. (2018). Hodgkin Lymphoma-Derived Extracellular Vesicles Change the Secretome of Fibroblasts Toward a CAF Phenotype. Front. Immunol..

[89] Metcalf D. (1989). The molecular control of cell division, differentiation commitment and maturation in haemopoietic cells. Nature.

[90] Alves J.J.P., De Medeiros Fernandes T.A.A., De Araujo J.M.G., Cobucci R.N.O., Lanza D.C.F., Bezerra F.L., Andrade V.S., Fernandes J.V. (2018). Th17 response in patients with cervical cancer. Oncol. Lett..

[91] Patil R.S., Shah S.U., Shrikhande S.V., Goel M., Dikshit R.P., Chiplunkar S.V. (2016). IL17 producing gammadeltaT cells induce angiogenesis and are associated with poor survival in gallbladder cancer patients. Int. J. Cancer.

[92] Shrivastava R., Asif M., Singh V., Dubey P., Ahmad Malik S., Lone M.U., Tewari B.N., Baghel K.S., Pal S., Nagar G.K. (2018). M2 polarization of macrophages by Oncostatin M in hypoxic tumor microenvironment is mediated by mTORC2 and promotes tumor growth and metastasis. Cytokine.

[93] Borregaard N., Cowland J.B. (1997). Granules of the human neutrophilic polymorphonuclear leukocyte. Blood.

[94] Felix K., Gaida M.M. (2016). Neutrophil-Derived Proteases in the Microenvironment of Pancreatic Cancer -Active Players in Tumor Progression. Int. J. Biol. Sci..

[95] El Rayes T., Catena R., Lee S., Stawowczyk M., Joshi N., Fischbach C., Powell C.A., Dannenberg A.J., Altorki N.K., Gao D. (2015). Lung inflammation promotes metastasis through neutrophil protease-mediated degradation of Tsp-1. Proc. Natl. Acad. Sci. USA.

[96] Okada Y. (2017). Kelley and Firestein’s Textbook of Rheumatology (Tenth Edition).

[97] Yui S., Osawa Y., Ichisugi T., Morimoto-Kamata R. (2014). Neutrophil cathepsin G, but not elastase, induces aggregation of MCF-7 mammary carcinoma cells by a protease activity-dependent cell-oriented mechanism. Mediat. Inflamm..

[98] Morimoto-Kamata R., Yui S. (2017). Insulin-like growth factor-1 signaling is responsible for cathepsin G-induced aggregation of breast cancer MCF-7 cells. Cancer Sci..

[99] Wilson T.J., Nannuru K.C., Futakuchi M., Sadanandam A., Singh R.K. (2008). Cathepsin G enhances mammary tumor-induced osteolysis by generating soluble receptor activator of nuclear factor-kappaB ligand. Cancer Res..

[100] DiCamillo S.J., Yang S., Panchenko M.V., Toselli P.A., Naggar E.F., Rich C.B., Stone P.J., Nugent M.A., Panchenko M.P. (2006). Neutrophil elastase-initiated EGFR/MEK/ERK signaling counteracts stabilizing effect of autocrine TGF-β on tropoelastin mRNA in lung fibroblasts. Am. J. Physiol. Lung Cell Mol. Physiol..

[101] Yang R., Zhong L., Yang X.Q., Jiang K.L., Li L., Song H., Liu B.Z. (2016). Neutrophil elastase enhances the proliferation and decreases apoptosis of leukemia cells via activation of PI3K/Akt signaling. Mol. Med. Rep..

[102] Wada Y., Yoshida K., Hihara J., Konishi K., Tanabe K., Ukon K., Taomoto J., Suzuki T., Mizuiri H. (2006). Sivelestat, a specific neutrophil elastase inhibitor, suppresses the growth of gastric carcinoma cells by preventing the release of transforming growth factor-alpha. Cancer Sci..

[103] Lerman I., Hammes S.R. (2018). Neutrophil elastase in the tumor microenvironment. Steroids.

[104] Lerman I., Garcia-Hernandez M.L., Rangel-Moreno J., Chiriboga L., Pan C., Nastiuk K.L., Krolewski J.J., Sen A., Hammes S.R. (2017). Infiltrating Myeloid Cells Exert Protumorigenic Actions via Neutrophil Elastase. Mol. Cancer Res..

[105] Kerros C., Tripathi S.C., Zha D., Mehrens J.M., Sergeeva A., Philips A.V., Qiao N., Peters H.L., Katayama H., Sukhumalchandra P. (2017). Neuropilin-1 mediates neutrophil elastase uptake and cross-presentation in breast cancer cells. J. Biol. Chem..

[106] Caruso J.A., Hunt K.K., Keyomarsi K. (2010). The neutrophil elastase inhibitor elafin triggers rb-mediated growth arrest and caspase-dependent apoptosis in breast cancer. Cancer Res..

[107] Akizuki M., Fukutomi T., Takasugi M., Takahashi S., Sato T., Harao M., Mizumoto T., Yamashita J. (2007). Prognostic significance of immunoreactive neutrophil elastase in human breast cancer: Long-term follow-up results in 313 patients. Neoplasia.

[108] Ho A.S., Chen C.H., Cheng C.C., Wang C.C., Lin H.C., Luo T.Y., Lien G.S., Chang J. (2014). Neutrophil elastase as a diagnostic marker and therapeutic target in colorectal cancers. Oncotarget.

[109] Gregory A.D., Houghton A.M. (2011). Tumor-associated neutrophils: New targets for cancer therapy. Cancer Res..

[110] Nagase H., Visse R., Murphy G. (2006). Structure and function of matrix metalloproteinases and TIMPs. Cardiovasc. Res..

[111] Chakrabarti S., Zee J.M., Patel K.D. (2006). Regulation of matrix metalloproteinase-9 (MMP-9) in TNF-stimulated neutrophils: Novel pathways for tertiary granule release. J. Leukoc. Biol..

[112] Gordon G.M., Ledee D.R., Feuer W.J., Fini M.E. (2009). Cytokines and signaling pathways regulating matrix metalloproteinase-9 (MMP-9) expression in corneal epithelial cells. J. Cell. Physiol..

[113] Hollborn M., Stathopoulos C., Steffen A., Wiedemann P., Kohen L., Bringmann A. (2007). Positive feedback regulation between MMP-9 and VEGF in human RPE cells. Investig. Ophthalmol. Vis. Sci..

[114] Pal-Ghosh S., Blanco T., Tadvalkar G., Pajoohesh-Ganji A., Parthasarathy A., Zieske J.D., Stepp M.A. (2011). MMP9 cleavage of the beta4 integrin ectodomain leads to recurrent epithelial erosions in mice. J. Cell Sci..

[115] Kobayashi T., Kim H., Liu X., Sugiura H., Kohyama T., Fang Q., Wen F.Q., Abe S., Wang X., Atkinson J.J. (2014). Matrix metalloproteinase-9 activates TGF-β and stimulates fibroblast contraction of collagen gels. Am. J. Physiol. Lung Cell. Mol. Physiol..

[116] Li H., Qiu Z., Li F., Wang C. (2017). The relationship between MMP-2 and MMP-9 expression levels with breast cancer incidence and prognosis. Oncol. Lett..

[117] Mehner C., Hockla A., Miller E., Ran S., Radisky D.C., Radisky E.S. (2014). Tumor cell-produced matrix metalloproteinase 9 (MMP-9) drives malignant progression and metastasis of basal-like triple negative breast cancer. Oncotarget.

[118] Yousef E.M., Tahir M.R., St-Pierre Y., Gaboury L.A. (2014). MMP-9 expression varies according to molecular subtypes of breast cancer. BMC Cancer.

[119] Pellikainen J.M., Ropponen K.M., Kataja V.V., Kellokoski J.K., Eskelinen M.J., Kosma V.M. (2004). Expression of matrix metalloproteinase (MMP)-2 and MMP-9 in breast cancer with a special reference to activator protein-2, HER2, and prognosis. Clin. Cancer Res..

[120] Brinkmann V., Reichard U., Goosmann C., Fauler B., Uhlemann Y., Weiss D.S., Weinrauch Y., Zychlinsky A. (2004). Neutrophil extracellular traps kill bacteria. Science.

[121] Fuchs T.A., Abed U., Goosmann C., Hurwitz R., Schulze I., Wahn V., Weinrauch Y., Brinkmann V., Zychlinsky A. (2007). Novel cell death program leads to neutrophil extracellular traps. J. Cell Biol..

[122] Erpenbeck L., Schon M.P. (2017). Neutrophil extracellular traps: Protagonists of cancer progression?. Oncogene.

[123] Pilsczek F.H., Salina D., Poon K.K., Fahey C., Yipp B.G., Sibley C.D., Robbins S.M., Green F.H., Surette M.G., Sugai M. (2010). A novel mechanism of rapid nuclear neutrophil extracellular trap formation in response to Staphylococcus aureus. J. Immunol..

[124] Yousefi S., Mihalache C., Kozlowski E., Schmid I., Simon H.U. (2009). Viable neutrophils release mitochondrial DNA to form neutrophil extracellular traps. Cell Death Differ..

[125] Berger-Achituv S., Brinkmann V., Abed U.A., Kuhn L.I., Ben-Ezra J., Elhasid R., Zychlinsky A. (2013). A proposed role for neutrophil extracellular traps in cancer immunoediting. Front. Immunol..

[126] Van der Windt D.J., Sud V., Zhang H., Varley P.R., Goswami J., Yazdani H.O., Tohme S., Loughran P., O’Doherty R.M., Minervini M.I. (2018). Neutrophil extracellular traps promote inflammation and development of hepatocellular carcinoma in nonalcoholic steatohepatitis. Hepatology.

[127] Tohme S., Yazdani H.O., Al-Khafaji A.B., Chidi A.P., Loughran P., Mowen K., Wang Y., Simmons R.L., Huang H., Tsung A. (2016). Neutrophil Extracellular Traps Promote the Development and Progression of Liver Metastases after Surgical Stress. Cancer Res..

[128] Sangaletti S., Tripodo C., Vitali C., Portararo P., Guarnotta C., Casalini P., Cappetti B., Miotti S., Pinciroli P., Fuligni F. (2014). Defective stromal remodeling and neutrophil extracellular traps in lymphoid tissues favor the transition from autoimmunity to lymphoma. Cancer Discov..

[129] Cools-Lartigue J., Spicer J., McDonald B., Gowing S., Chow S., Giannias B., Bourdeau F., Kubes P., Ferri L. (2013). Neutrophil extracellular traps sequester circulating tumor cells and promote metastasis. J. Clin. Investig..

[130] Najmeh S., Cools-Lartigue J., Rayes R.F., Gowing S., Vourtzoumis P., Bourdeau F., Giannias B., Berube J., Rousseau S., Ferri L.E. (2017). Neutrophil extracellular traps sequester circulating tumor cells via beta1-integrin mediated interactions. Int. J. Cancer.

[131] Oklu R., Sheth R.A., Wong K.H.K., Jahromi A.H., Albadawi H. (2017). Neutrophil extracellular traps are increased in cancer patients but does not associate with venous thrombosis. Cardiovasc. Diagn. Ther..

[132] Richardson J.J.R., Hendrickse C., Gao-Smith F., Thickett D.R. (2017). Neutrophil Extracellular Trap Production in Patients with Colorectal Cancer In Vitro. Int. J. Inflam..

[133] Albrengues J., Shields M.A., Ng D., Park C.G., Ambrico A., Poindexter M.E., Upadhyay P., Uyeminami D.L., Pommier A., Kuttner V. (2018). Neutrophil extracellular traps produced during inflammation awaken dormant cancer cells in mice. Science.

[134] Park J., Wysocki R.W., Amoozgar Z., Maiorino L., Fein M.R., Jorns J., Schott A.F., Kinugasa-Katayama Y., Lee Y., Won N.H. (2016). Cancer cells induce metastasis-supporting neutrophil extracellular DNA traps. Sci. Transl. Med..

[135] Son B., Lee S., Youn H., Kim E., Kim W., Youn B. (2017). The role of tumor microenvironment in therapeutic resistance. Oncotarget.

[136] Leibowitz-Amit R., Templeton A.J., Omlin A., Pezaro C., Atenafu E.G., Keizman D., Vera-Badillo F., Seah J.A., Attard G., Knox J.J. (2014). Clinical variables associated with PSA response to abiraterone acetate in patients with metastatic castration-resistant prostate cancer. Ann. Oncol..

[137] Nywening T.M., Belt B.A., Cullinan D.R., Panni R.Z., Han B.J., Sanford D.E., Jacobs R.C., Ye J., Patel A.A., Gillanders W.E. (2018). Targeting both tumour-associated CXCR2(+) neutrophils and CCR2(+) macrophages disrupts myeloid recruitment and improves chemotherapeutic responses in pancreatic ductal adenocarcinoma. Gut.

[138] Finke J., Ko J., Rini B., Rayman P., Ireland J., Cohen P. (2011). MDSC as a mechanism of tumor escape from sunitinib mediated anti-angiogenic therapy. Int. Immunopharmacol..

[139] Foekens J.A., Ries C., Look M.P., Gippner-Steppert C., Klijn J.G., Jochum M. (2003). Elevated expression of polymorphonuclear leukocyte elastase in breast cancer tissue is associated with tamoxifen failure in patients with advanced disease. Br. J. Cancer.

[140] Elaskalani O., Razak N.B., Falasca M., Metharom P. (2017). Epithelial-mesenchymal transition as a therapeutic target for overcoming chemoresistance in pancreatic cancer. World J. Gastrointest. Oncol..

[141] Schiffmann L.M., Fritsch M., Gebauer F., Gunther S.D., Stair N.R., Seeger J.M., Thangarajah F., Dieplinger G., Bludau M., Alakus H. (2019). Tumour-infiltrating neutrophils counteract anti-VEGF therapy in metastatic colorectal cancer. Br. J. Cancer.

[142] Chung A.S., Wu X., Zhuang G., Ngu H., Kasman I., Zhang J., Vernes J.M., Jiang Z., Meng Y.G., Peale F.V. (2013). An interleukin-17-mediated paracrine network promotes tumor resistance to anti-angiogenic therapy. Nat. Med..

[143] Gershkovitz M., Fainsod-Levi T., Zelter T., Sionov R.V., Granot Z. (2019). TRPM2 modulates neutrophil attraction to murine tumor cells by regulating CXCL2 expression. Cancer Immunol. Immunother..

[144] Sun B., Qin W., Song M., Liu L., Yu Y., Qi X., Sun H. (2018). Neutrophil Suppresses Tumor Cell Proliferation via Fas /Fas Ligand Pathway Mediated Cell Cycle Arrested. Int. J. Biol. Sci..

[145] Finisguerra V., Di Conza G., Di Matteo M., Serneels J., Costa S., Thompson A.A., Wauters E., Walmsley S., Prenen H., Granot Z. (2015). MET is required for the recruitment of anti-tumoural neutrophils. Nature.

[146] Chang C.Y., Tai J.A., Li S., Nishikawa T., Kaneda Y. (2016). Virus-stimulated neutrophils in the tumor microenvironment enhance T cell-mediated anti-tumor immunity. Oncotarget.

[147] Lin M., Jackson P., Tester A.M., Diaconu E., Overall C.M., Blalock J.E., Pearlman E. (2008). Matrix metalloproteinase-8 facilitates neutrophil migration through the corneal stromal matrix by collagen degradation and production of the chemotactic peptide Pro-Gly-Pro. Am. J. Pathol..

[148] Gutierrez-Fernandez A., Fueyo A., Folgueras A.R., Garabaya C., Pennington C.J., Pilgrim S., Edwards D.R., Holliday D.L., Jones J.L., Span P.N. (2008). Matrix metalloproteinase-8 functions as a metastasis suppressor through modulation of tumor cell adhesion and invasion. Cancer Res..

[149] Thirkettle S., Decock J., Arnold H., Pennington C.J., Jaworski D.M., Edwards D.R. (2013). Matrix metalloproteinase 8 (collagenase 2) induces the expression of interleukins 6 and 8 in breast cancer cells. J. Biol. Chem..

[150] Bockelman C., Beilmann-Lehtonen I., Kaprio T., Koskensalo S., Tervahartiala T., Mustonen H., Stenman U.H., Sorsa T., Haglund C. (2018). Serum MMP-8 and TIMP-1 predict prognosis in colorectal cancer. BMC Cancer.

[151] Quigley J.P., Deryugina E.I. (2012). Combating angiogenesis early: Potential of targeting tumor-recruited neutrophils in cancer therapy. Futur. Oncol..

[152] Gargiulo P., Dietrich D., Herrmann R., Bodoky G., Ruhstaller T., Scheithauer W., Glimelius B., Berardi S., Pignata S., Brauchli P. (2019). Predicting mortality and adverse events in patients with advanced pancreatic cancer treated with palliative gemcitabine-based chemotherapy in a multicentre phase III randomized clinical trial: The APC-SAKK risk scores. Ther. Adv. Med. Oncol..

[153] Cheng C.C., Chang J., Chen L.Y., Ho A.S., Huang K.J., Lee S.C., Mai F.D., Chang C.C. (2012). Human Neutrophil peptides 1–3 as gastric cancer tissue markers measured by MALDI-imaging mass spectrometry: Implications for infiltrated neutrophils as a tumor target. Dis. Markers.

[154] Gurluler E., Tumay L.V., Guner O.S., Kucukmetin N.T., Hizli B., Zorluoglu A. (2014). Oncostatin-M as a novel biomarker in colon cancer patients and its association with clinicopathologic variables. Eur. Rev. Med. Pharmacol. Sci..

[155] Granot Z., Jablonska J. (2015). Distinct Functions of Neutrophil in Cancer and Its Regulation. Mediat. Inflamm..

[156] De Soyza A., Pavord I., Elborn J.S., Smith D., Wray H., Puu M., Larsson B., Stockley R. (2015). A randomized, placebo-controlled study of the CXCR2 antagonist AZD5069 in bronchiectasis. Eur. Respir. J..

[157] Suda K., Kitagawa Y., Ozawa S., Miyasho T., Okamoto M., Saikawa Y., Ueda M., Yamada S., Tasaka S., Funakoshi Y. (2007). Neutrophil elastase inhibitor improves postoperative clinical courses after thoracic esophagectomy. Dis. Esophagus.

